# Recurrent monocular exudative retinal detachment as the first manifestation of squamous cell lung cancer: A case report

**DOI:** 10.1097/MD.0000000000025189

**Published:** 2021-03-19

**Authors:** Mateusz Zarzecki, Emil Saeed, Zofia Mariak, Joanna Konopińska

**Affiliations:** Department of Ophthalmology, Medical University of Bialystok, Poland.

**Keywords:** cancer, exudative retinal detachment, oncology, retina, squamous cell lung cancer

## Abstract

**Rationale:**

In this report, we present an extremely rare case of recurrent monocular exudative retinal detachment without concomitant ocular metastases. This turned out to be the first symptom of squamous cell lung cancer.

**Patient concerns:**

A 63-year-old woman was referred to our ophthalmology clinic by her primary care physician with a complaint of deteriorating vision in her right eye that had started four months prior, without concomitant pain.

**Diagnoses:**

We observed a detachment in the lower part of the retina during her ophthalmoscopy. We did not find any tears, holes, or degenerative changes in the periphery of the retina of the right eye during the surgery. In addition, plaques, tumor masses, and metastases were absent. Therefore, we diagnosed her with unilateral paraneoplastic exudative retinal detachment. Imaging tests performed before surgery revealed perihilar density with a visible air bronchogram in the middle field of the left lung. This turned out to be squamous cell carcinoma.

**Interventions:**

Patient underwent pars plana vitrectomy and routine laboratory and imaging tests before the procedure that utilized 20-gauge instrumentation. The subretinal fluid and was drained and a tamponade using Densiron (Fluoron Co, Neu-Ulm, Germany) was applied. After ophthalmic treatment, patient underwent complex oncological treatment based on chemotherapy and radiotherapy.

**Outcomes:**

Despite the application of heavy silicone oil (Densiron) into the vitreous chamber, we observed a recurrence of retinal detachment in the right eye during the follow-up visit, 13 months after the first ophthalmic surgery. Following subsequent pars plana vitrectomy, the Densiron and subretinal membranes were removed. Despite oncological treatment, the patient died, twenty months after the appearance of the first ocular symptoms.

**Lessons:**

Exudative retinal detachment without tumor metastasis to the eyeball can be one of the first signs of lung cancer in rare cases. Multidisciplinary care and imaging methods with greater accuracy will provide comprehensive care to the patients. It will not only facilitate timely detection and treatment of lung tumors but also for a plethora of oncological diseases.

## 1 Introduction

Lung cancer is the second most frequently diagnosed cancer after breast cancer in women, and prostate cancer in men.^[1]^ However, according to statistical reports, it is the main cause of cancer-related deaths around the world.^[2]^ Lung cancer is usually asymptomatic, and the initial signs appear late in the course of the disease. Therefore, the diagnostic process becomes extremely challenging. Surprisingly, the first symptoms may comprise ocular cancer-associated retinopathy, bilateral diffuse uveal melanocytic proliferation, paraneoplastic retinopathies (i.e., paraneoplastic vitelliform maculopathy), acute exudative polymorphous vitelliform maculopathy, or best macular dystrophy in rare cases.^[3]^

There are approximately 50 reported cases worldwide with exudative retinal detachment as the first symptom of lung cancer metastasis to the eyeball.^[4–8]^ According to these studies, it is more common among men aged 55.1 ± 11.2 years and former or active smokers. The tumor is usually an adenocarcinoma, which mainly affects the left eye. In addition, the upper left lobe is usually the primary origin.^[7]^ However, only two cases of exudative retinal detachment without concomitant ocular metastasis as the first symptom of lung cancer have been documented worldwide.^[9,10]^ In addition, both were related to adenocarcinoma. In this report, we present an extremely rare case of recurrent monocular exudative retinal detachment in the right eye, without concomitant ocular metastasis, as the first symptom of squamous cell lung cancer.

## 2 Case presentation

**Figure 1:**
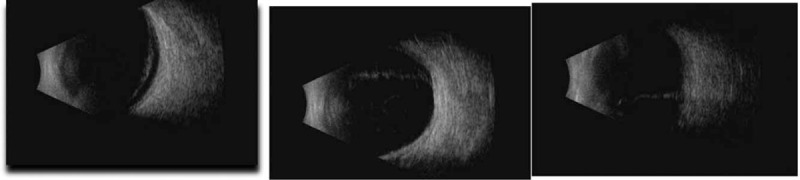
Flat retinal detachment covering the lower hemisphere of the eye.

A 63-year-old woman was referred by her general practitioner to our ophthalmology clinic in April 2018 with a complaint of deteriorating vision in her right eye that had started 4 months prior, without any concomitant pain. She complained of gradual deterioration of her vision and the presence of an “eye curtain” in the upper region of her visual field. She reported having a high myopia (−7 diopters [D]) from an early age. The best-corrected visual acuity (BCVA) of the right eye was 0.1 (measured with a Snellen's chart) with normal light projection. In contrast, it was 0.5 for the left eye. We used a Goldman Applanation Tonometer to measure the intraocular pressure, which was 16 and 19 mm Hg in the right and left eye, respectively. A biomicroscopic examination of the right eye revealed signs of anterior uveitis, namely posterior synechiae, corneal deposits, and early nuclear cataract. However, the anterior fluid chamber did not show signs of tyndallization. We also found early nuclear cataracts in the left eye. An examination of the fundus through a narrow pupil (no possibility of mydriasis due to annular synechia) revealed a normal optic disc, a macula with no reflex, and possible detachment of the lower part of the retina. The retinal detachment was later confirmed using A/B-mode ultrasound v 5.0.0 scan of the eyeball (Aviso S, Quantel Medical, Paris, France) (Fig. 1). The retina in the area available for assessment was free of focal lesions and there was no shifting fluid note on B scan. Neither signs of tumor nor any abnormalities were observed in the fundus of the left eye. At that time, we did not suspected exudative retinal detachment, and we qualified the patient for surgery surgical treatment.

The medical history of the patient showed that she underwent Freund's resection of the uterus and appendages, appendectomy, and resection of the greater omentum for the treatment of G2 ovarian endometrioid cystoadenocarcinoma in 2012. She was referred to the ophthalmology department for emergency surgery and was admitted the next day. She was examined preoperatively and scheduled for pars plana vitrectomy . In addition, she underwent routine pre-operative laboratory and imaging tests that utilized 20-gauge instrumentation. We separated the posterior annular synechia and removed the lens with an artificial one-piece intraocular lens implantation (+17 D) into the capsular bag (Akreos ADAPT, Bausch & Lomb, Rochester, USA). A retinal endolaser was used during the procedure. We drained the subretinal fluid and applied a tamponade using Densiron (Fluoron Co, Neu-Ulm, Germany). The patient was ultimately diagnosed with unilateral paraneoplastic exudative retinal detachment.

**Figure 2:**
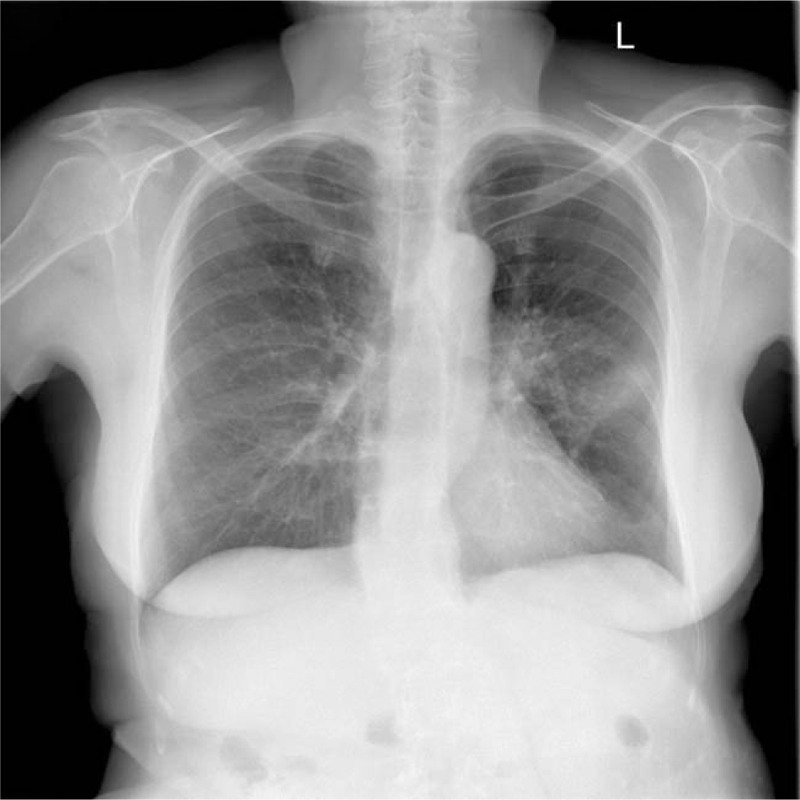
X-ray of the patient's lungs before the surgery, showing perihilar density with a visible air bronchogram in the middle field of the left lung.

Chest radiography revealed perihilar radiodensity with a visible air bronchogram in the middle field of the left lung (Fig. 2). In addition, a contrast enhanced-computed tomography (CT) scan of the chest revealed a nodular thyroid and a tumorous lesion in the upper-middle part of the left lung hilum, 33 × 30 mm in size. We also identified a 24 × 12 mm lesion and numerous smaller lesions of a small spot-nodular character in the third segmented bronchus. The mediastinum revealed enlarged lymph nodes ranging from 9 to 17 mm in size. Furthermore, ultrasound scans of the abdomen and retroperitoneal space showed first-degree steatosis of the liver.

Results of laboratory testing were as follows: an increased prothrombin time (PT) (140%), slightly shortened activated partial thromboplastin time (APTT) (23.7 s), increased fibrinogen level (416 mg/dL), slightly increased C-reactive protein level (10.2 mg/L), and a history of toxoplasmosis and human herpesvirus 5 infection (presence of immunoglobulin G antibodies). We ruled out the possibility of an active infection based on these findings. In addition, the blood tests revealed a reduction in the level of thyroid-stimulating hormone. Thus, she was referred for an endocrine consultation. This resulted in the diagnosis of subclinical hyperthyroidism with nodular goiter and recommendation for further treatment at the outpatient endocrine clinic.

Considering the nodular changes detected in the lungs, we conducted thoracic and pulmonary consultations. We suspected the presence of a malignant tumor and referred the patient to the pulmonary disease department for further examination and management.

During her stay in the ophthalmology ward, we observed an effusion in the anterior chamber and a grade 1+ flare, indicating postoperative inflammatory reaction of the anterior uveal segment. Postoperative treatment included the intravenous administration of steroids (8 mg dexamethasone twice daily) and topical administration of a combination of steroids, antibiotics, and mydriatics to the right eye (one drop each of dexamethasone, moxifloxacin, and 1% tropicamide four times daily for 7 days). Dexamethasone dose was later tapered over 4 weeks. We used a neodymium-doped yttrium aluminum garnet laser to cut the exudative membrane from the bottom. When the fundus could be visualized, we performed fluorescent angiography, indocyanine green angiography, and enhanced MRI. The aforementioned examinations failed to reveal a tumor mass. She was then discharged with recommendations for further general and local treatments.

The patient was admitted to the pulmonary disease ward on June 7, 2018 and underwent bronchoscopy. The examination revealed a smooth, shiny cauliflower-like lesion that completely enclosed the bronchial lumen of the 1st, 2nd, and 3rd segments. We performed transthoracic fine-needle aspiration cytology under CT to collect the sample for histopathological examination. She was also sent for an endobronchial ultrasound-guided transbronchial needle aspiration examination of the mediastinal lymph nodes and assessment of thymidine kinase expression using positron emission tomography (PET).

A PET-CT examination, performed on July 4, 2018, revealed a 33 × 26 mm tumor with increased fluorodeoxyglucose metabolism in the left lung hilum. The latter was connected to the left pulmonary artery and surrounded the main bronchus and upper lobe bronchus. Additionally, we observed an enlargement of the paratracheal lymph nodes and the aorto-pulmonary window up to 12 mm. The patient was deemed ineligible for surgery based on the results from the PET-CT scan and previous tests. Results of pathomorphological analyses and imaging studies led to a diagnosis of squamous cell carcinoma of the left lung (T4N2M0).

She was admitted to the oncology center for chemotherapy on September 5, 2018. She was administered 100 mg vinorelbine (Navelbine) in a divided dose (two 20 mg tablets and two 30 mg tablets) and 60 mg cisplatin with a recommendation to report to the clinic for future chemotherapy.

Despite the hepatoprotective treatment, we observed an increase in the transaminase levels after the second part of the first course of chemotherapy. This, in turn, delayed the administration of the next cycle after exclusion of the potential non-drug causes.

The tumor stage regressed to T2N2M0 (tumor size, 12 × 7 mm), and the lymph node in the aorto-pulmonary window reduced to 11 mm after the treatment. This resulted in a decision to implement radical intensity-modulated radiation therapy on the tumor area in the left lung, later extended to include groups IV and V of the lymph nodes.

High anisometropia led to the recommendation for a cataract phacoemulsification procedure with implantation of an artificial intraocular lens (+13 D) (Acreos Adapt, Bausch & Lomb, Rochester, USA) in the left eye. This procedure was performed in December 2018, followed by a BCVA of 1.0, two weeks after the surgery.

The early imaging tests did not reveal any alarming results. However, the disease progressed with time. While the tumor enlarged to a size of 27 × 11 mm, the lymph node in the aorto-pulmonary window enlarged to 13 mm. This was concomitant with a meta change with lysis features at the bronchovascular bundle that penetrated the pleura of the right lung. Thus, the relapse qualified the patient for treatment with pembrolizumab.

**Figure 3:**
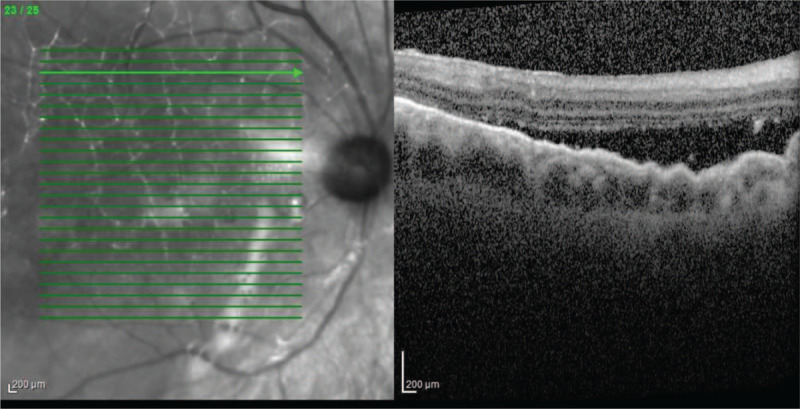
The presence of fluid between the neurosensory retina and the pigmented layer.

**Figure 4:**
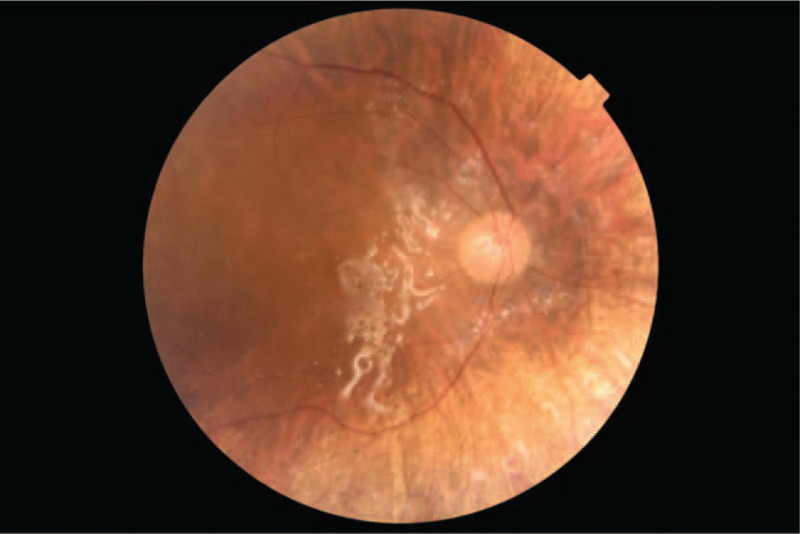
Visible tamponade of the vitreous chamber using Densiron.

Despite the administration of heavy silicone oil (Densiron) into the vitreous chamber, we observed fluid under the neurosensory retina and a recurrence of retinal detachment in the right eye during the follow-up visit, 13 months after the first ophthalmic surgery (Figs. 3 and 4). OCT picture showed significant diffuse RPE undulations, as it is common to have these changes are common in exudative retinal detachment. The BCVA of the right eye was 0.4. Following pars plana vitrectomy, the Densiron and subretinal membranes were removed. This was followed by the application of a Siluron 5000 (Fluoron GmbH, Ulm, Germany) tamponade. Intraoperative evaluation of the retina did not reveal any metastatic changes. Postoperative treatment included instillation of an antibiotic and a steroid into the right eye four times daily for 14 days (moxifloxacin and dexamethasone). In addition to a normal healing process, the BCVA of the right eye was 0.6 with a +4.0 D correction the following week.

The patient underwent a CT scan of the central nervous system (with and without contrast) during chemotherapy. The results showed meta changes in the left frontal lobe and on the border of the frontal and parietal lobes on the left side, apart from infiltration of the bone structures. While there was massive osteolysis in the right frontal bone, the mandibular head on the right side showed signs of dilatation and partial osteolysis. She underwent a second course of treatment, after which the meta-type lesions continued to progress and the tumor progressed to T2N2M1.

Despite treatment, the patient died on January 1, 2020, twenty months after the appearance of the first ocular symptoms.

## 3 Discussion

This is the third reported case of exudative retinal detachment without concomitant ocular metastasis preceding the diagnosis of a neoplastic process. Unlike the other cases,^[9,10]^ our patient had no history of smoking and was diagnosed with squamous cell carcinoma.

Burgess et al reported a case of bilateral paraneoplastic exudative retinal detachment associated with adenocarcinoma of the lung in a 47-year-old man. He was a smoker with ophthalmic symptoms as the first manifestation of a general disease.^[9]^ He underwent vitrectomies of both eyes combined with a silicone oil tamponade and subretinal fluid drainage. However, there was no metastasis to the eyeball both during surgery and in the later stages of the disease. An adenocarcinoma is typically localized to the peripheral parts of the lungs. However, the chest CT scan of the patient described above, showed enlarged pre-tracheal, right hilar, and subcarinal lymph nodes, together with a calcific focus in the right upper lobe of the lung.

Modrzejewska et al. described the case of a 50-year-old woman with exudative retinal detachment as the first symptom of disseminated adenocarcinoma of the lung.^[10]^ Considering the advancement of the neoplastic process and metastasis to the central nervous system (T2N3M1), she underwent four cycles of paclitaxel-carboplatin palliative chemotherapy and left lung radiotherapy. However, she died four months after the appearance of the first ophthalmic symptoms. Centrally located lesions were radiologically visualized apart from the thick atelectasis of the entire superior lobe and the lower part of the inferior lobe of the left lung in this case. In addition, there were signs of enlarged lymph nodes within the pericarinal area and the right pulmonary hilum.^[10]^ The location of the lesion in our case was similar to that reported by Modrzejewska et al. and Burgess et al. The nodular lesion was located in the upper-middle part of the left lung hilum, adjacent to the division of the pulmonary artery and the upper lobe artery, a location typical of squamous cell carcinoma. Thus, the localization of the cancer mass is common in these three cases.

The pathomechanism of exudative retinal detachment is yet to be completely understood. The theory of the pathogenesis of exudative retinal detachment sheds light on disturbances in the physiology of cells that build the blood-retinal barrier due to insufficient blood supply, thus causing hypoxia. It may be the result of a variety of diseases, usually systemic disorders, including chronic inflammation, infections, defects in the blood vessels, degenerative states, or cancer.^[11]^ The most common cause of exudative retinal detachment is neoplastic metastases to various eye structures, most commonly the vascular part, due to cancer.^[5,12,13]^ According to case reports, lung cancer in men and breast cancer in women are the main sources of metastases to the visual structures. Moreover, metastases may originate from cancer of the prostate, thyroid, digestive system, kidneys, and the lymphatic system.^[14]^ However, no specific cause of the condition has been identified to date.

Numerous theories discuss dysfunction within the cells of the blood-retinal barrier, caused by the circulation of cancer cells in the bloodstream. This eventually results in leakage of fluid into the subretinal space. Others report on disorders in the coagulation system and abnormalities in the morphology of peripheral blood vessels, which may result in the formation of thrombotic plugs, thus causing ischemia and hypoxia.^[15]^ This, in turn, is confirmed by the results of the laboratory tests conducted on our patient, which revealed an increased PT, a slightly shortened APTT, and an increased fibrinogen level. Modrzejewska et al. reported abnormal erythrocyte sedimentation rates and elevated levels of D-dimers and fibrinogen.^[10]^ In contrast, Burgess et al. identified an increase in plasma viscosity as the only abnormality.^[9]^ These changes are typical of metastatic neoplastic processes and may trigger retinal ischemia, increased permeability of the vascular endothelium, and subretinal fluid leakage. Myopia and increased axial length (25.4 mm) in our patient might have contributed to the weak connections between the epithelial cells.

Apart from surgical treatment, the treatment of exudative retinal detachment is based on eliminating its primary cause. Chemotherapy, radiotherapy, hormone therapy, immunotherapy, or palliative treatment can be used as the last resort for the management of neoplastic disease, depending on the extent and number of organs affected by metastasis.^[16–18]^ However, our case report indicated that exudative retinal detachment may occur without metastasis and is visible on imaging tests. Thus, gamma globulin, plasmapheresis, and interleukin-2 receptor blockade, with a specific monoclonal antibody is the proposed first-line treatment in such cases.^[9]^ We started with surgical treatment in this case, owing to the difficulty in the accurate assessment of the retinal circumference in terms of retinal tear. This could be attributed to the presence of annular synechia. Long-term retinal detachment can also lead to chronic inflammation of the anterior uveal segment.^[19]^ However, we were unable to determine which of these conditions were primary and secondary, prior to the surgery. Despite the surgery, the retinal detachment recurred and the final BCVA was 0.6. Nonetheless, we could not achieve sustained anatomical success.

We had doubts about the association between the changes in the eye to those in the lungs, and whether the lung tumor was the primary origin. Since the patient was diagnosed with ovarian cancer in 2012, we also considered that this retinal detachment could be attributed to the adenocystic carcinoma. Cancers of the genitourinary system are also known to cause paraneoplastic syndrome apart from lung cancer. However, the previous tumor was determined to be in the early stage. This, in turn, was confirmed by the histopathological characteristics of the intraoperative fragments, which showed no infiltration or other features of metastasis. Therefore, such a radical surgical procedure that the patient underwent with a margin of healthy tissues might result in complete recovery. Moreover, the cancer found in the ovary was an endometrial cystadenocarcinoma. Thus, it is extremely unlikely to manifest as squamous cell carcinoma in the lung more than five years after the surgery. Both these tumors have different histological origins. While the tumor comprises cystic-glandular endometrial cells in the ovary, it consists of squamous cells when occurring in the lungs. This observation is also supported by the normal CA 125 marker levels one year after hysterectomy. Thus, the tumor was manifested by predisposition rather than the pulmonary recurrence of the previous cancer in this case.

The limitations of our case report include a lack of accurate information on the history of neoplasms in the patient's family, a lack of genetic tests that facilitate an evaluation of the relationship between individual neoplastic diseases, and a lack of tested human leukocyte antigens (HLA) (considering the correlation between diseases associated with exudative retinal detachment and HLA).^[20]^ We also failed to obtain subretinal fluid or a vitreous tap for histological evaluation. We are not aware of whether tumor cells were present or not, and this is fundamental to rule out metastasis. However, at the time of surgery, we did not suspect exudative retinal detachment, and we suspected rhegmatogenous retinal detachment with secondary uveitis. Additionally, we did not perform an ocular post-mortem analysis of the eyes, when the patient expired. Despite these limitations, the case described in this report remains extremely rare. We gradually uncovered and evaluated the individual components of the diagnosis. Unfortunately, the patient died prior to a complete analysis of the disease.

Exudative retinal detachment that occurs during the course of paraneoplastic syndrome is not always concomitant with tumor metastasis to the eyeball. Patients with neoplastic disease require an interdisciplinary approach to treatment. This permits physicians to arrive at appropriate diagnoses and provide comprehensive and effective care.

## Acknowledgments

The authors thank Editage (www.editage.com) for their English language editing service.

## Author contributions

**Conceptualization:** Joanna Konopinska.

**Investigation:** Mateusz Zarzecki, Emil Saeed, Joanna Konopinska.

**Methodology:** Emil Saeed, Joanna Konopinska.

**Project administration:** Zofia Mariak.

**Resources:** Mateusz Zarzecki.

**Supervision:** Zofia Mariak, Joanna Konopinska.

**Validation:** Joanna Konopinska.

**Visualization:** Joanna Konopinska.

**Writing – original draft:** Mateusz Zarzecki, Joanna Konopinska.

**Writing – review & editing:** Emil Saeed, Zofia Mariak, Joanna Konopinska.

## References

[1] de Groot PM Wu CC Carter BW. The epidemiology of lung cancer. Transl Lung Cancer Res 2018;7:220–33. 30050761 10.21037/tlcr.2018.05.06 PMC6037963

[2] National Collaborating Centre for Cancer (UK). The diagnosis and treatment of lung cancer (Update). Cardiff (UK). 2011. Available from: https://www.ncbi.nlm.nih.gov/books/NBK99043/22855970

[3] Arepalli S Kaliki S Shields CL. Choroidal metastases: origin, features, and therapy. Indian J Ophthalmol 2015;63:122–7. 25827542 10.4103/0301-4738.154380 PMC4399120

[4] Gloor BP Marmor MF. Controversy over the etiology and therapy of retinal detachment: the struggles of Jules Gonin. Surv Ophthalmol 2013;58:184–95. 23257154 10.1016/j.survophthal.2012.09.002

[5] Kopf B Ercolani D Zago S. Unilateral retinal detachment as the initial sign of lung adenocarcinoma. J Exp Clin Cancer Res 2007;26:141–3. 17550143

[6] Maturu VN Singh N Bansal P. Combination of intravitreal bevacizumab and systemic therapy for choroidal metastases from lung cancer: report of two cases and a systematic review of literature. Med Oncol 2014;31:901. 24604338 10.1007/s12032-014-0901-z

[7] Singh N Kulkarni P Aggarwal AN. Choroidal metastasis as a presenting manifestation of lung cancer: a report of 3 cases and systematic review of the literature. Medicine 2012;91:179–94. 22732948 10.1097/MD.0b013e3182574a0b

[8] Vicini G Nicolosi C Pieretti G. Large choroidal metastasis with exudative retinal detachment as presenting manifestation of small cell lung cancer: a case report. Respir Med Case Rep 2020;30:101074. 32420018 10.1016/j.rmcr.2020.101074 PMC7218149

[9] Burgess PI Kenawy N Pearce IA. Paraneoplastic exudative retinal detachment associated with adenocarcinoma of the lung. Eur J Ophthalmol 2010;20:952–4. 20213612 10.1177/112067211002000523

[10] Modrzejewska A Krzystolik K Lubiński W Modrzejewska M. Spontaneous unilateral exudative retinal detachment as the first manifestation of lung cancer. *Klinika Oczna*. 2018; 10;85–8.

[11] Dmuchowska DA Krasnicki P Mariak Z. Can optical coherence tomography replace fluorescein angiography in detection of ischemic diabetic maculopathy? Graefes Arch Clin Exp Ophthalmol 2014;252:731–8. 24292702 10.1007/s00417-013-2518-x PMC4007050

[12] Amer R Nalci H Yalçindağ N. Exudative retinal detachment. Surv Ophthalmol 2017;62:723–69. 28506603 10.1016/j.survophthal.2017.05.001

[13] Saha K Basuthakur S Jash D. Lung cancer presenting as visual impairment. South Asian J Cancer 2013;2:86. 10.4103/2278-330X.110501 PMC3876653 24455564

[14] John VJ Jacobson MS Grossniklaus HE. Bilateral choroidal metastasis as the presenting sign of small cell lung carcinoma. J Thorac Oncol 2010;5:1289. 20661088 10.1097/JTO.0b013e3181e004cd PMC2989178

[15] Lampaki S Kioumis I Pitsiou G. Lung cancer and eye metastases. Med Hypothesis Discov Innov Ophthalmol 2014;3:40–4. 25738158 PMC4346676

[16] Chinta S Rani PK Manusani U. Bilateral exudative retinal detachment as a presenting sign of acute lymphoblastic leukemia. Middle East Afr J Ophthalmol 2012;19:410–2. 23248545 10.4103/0974-9233.102762 PMC3519130

[17] Shimomura I Tada Y Miura G. Choroidal metastasis of non-small cell lung cancer that responded to gefitinib. Case Rep Ophthalmol Med 2013;2013:213124. 24109538 10.1155/2013/213124 PMC3787632

[18] Kreusel KM Wiegel T Stange M. Choroidal metastasis in disseminated lung cancer: frequency and risk factors. Am J Ophthalmol 2002;134:445–7. 12208262 10.1016/s0002-9394(02)01580-5

[19] Albert DM Rubenstein RA Scheie HG. Tumor metastasis to the eye. I. Incidence in 213 adult patients with generalized malignancy. Am J Ophthalmol 1967;63:723–6. 6022244

[20] Rabinowitz R Schneck M Levy J. Bilateral multifocal choroiditis with serous retinal detachment in a patient with Brucella infection: case report and review of the literature. Arch Ophthalmol 2005;123:116–8. 15642826 10.1001/archopht.123.1.116

